# In-Depth Investigation on Potential Mechanism of Forest-Grown Ginseng Alleviating Alzheimer’s Disease via UHPLC-MS-Based Metabolomics

**DOI:** 10.3390/metabo15020093

**Published:** 2025-02-03

**Authors:** Huazhou Niu, Meng Zhang, Kaiyue Zhang, Saibire Aishan, Hui Li, Wei Wu

**Affiliations:** Jilin Ginseng Academy, Changchun University of Chinese Medicine, Changchun 130117, China

**Keywords:** forest-grown ginseng, Alzheimer’s disease, UHPLC-MS, metabolites, neuroinflammation

## Abstract

Background: Alzheimer’s disease is a central nervous system degenerative disease closely related to age with a complex pathogenesis. As a natural medicinal plant, forest-grown ginseng (GSF) contains abundant ginsenosides and offers significant neuroprotective effects. Methods: In this study, we comprehensively investigated the effect of GSF on the cell viability of PC12 cells in an AD model alongside metabolic changes in the serum and brains of mice, combined with an efficacy evaluation of PC12 cells in vitro and UHPLC-MS-based metabolomics in vivo. The goal of this study is to clarify the potential mechanism of GSF in treating AD. Results: The PC12 cell results showed that GSF can promote the proliferation of PC12 cells, reduce the content of IL-8, increase the activity of SOD, and alleviate the inflammation and oxidative stress induced by Aβ25~35. The immunohistochemical results for the mouse brain tissue also showed that GSF could reduce the inflammatory response of mouse brain tissue by reducing the overexpression of IBa1. AD was alleviated by reducing Aβ protein deposition in the mouse brain tissue. An untargeted metabolomics analysis was performed using UHPLC-Q-Exactive MS and principal component analysis (PCA) to identify the differentially expressed metabolites in the serum and brain tissue of AD mice after treatment. Twenty and seventeen different metabolites were identified in the serum and brain tissue, respectively. The pathway enrichment analysis of differential metabolites showed that GSF could treat AD by up-regulating succinic acid semialdehyde, carbamoyl phosphate, Sphingosine 1-phosphate, L-cystathionine, 2-ketobutyric acid, Vanillylmandelic acid, and D-Ribose to regulate sphingomyelin metabolism, the synthesis and metabolism of neurotransmitters and precursors, and energy metabolism. Conclusions: GSF can reduce neuroinflammation and alleviate Alzheimer’s disease by regulating the metabolic disorders of amino acids, sphingolipids, unsaturated fatty acids, and arachidonic acid in mice serum and brain tissue metabolites. These results suggest a link between metabolite imbalance and AD, and reveal the basis for the mechanism of ginsenosides in AD treatment.

## 1. Introduction

Forest-grown ginseng (GSF, *Panax ginseng* C. A. Mey.) is an Araliaceae perennial herb. Ginsenosides are one of the main active ingredients in GSF and offer extensive pharmacological activities [1]. Unlike the growing environment of garden ginseng, GSF grows naturally without human intervention after being sown into a natural environment. Pharmacokinetic and pharmacometabolic studies have found that GSF has stronger antioxidant, anti-inflammatory, tumor-inhibiting, fatigue-relieving, and blood-pressure-lowering effects than garden ginseng. Some studies have shown that the ginsenosides in GSF mainly include ginsenosides Re, Rg1, Rf, Rg2, Rb1, Rc, Rb2, Rb3, Rd, Rg3, Rk1, and Rg5 [2]. As the main active components of GSF, ginsenosides exert extensive pharmacological activities, with GSF usually employed as a clinical drug. To date, a total of 620 ginsenosides have been successfully characterized in RRPG, including 309 proginsenosides, 258 proginsenosides, and 53 zipiperane ginsenosides [3]. Ginsenoside Rb1 can treat ischemic stroke by inhibiting the activation of astrocytes and promoting the transfer of astrocyte mitochondria to neurons [4]. Ginsenoside Rg3 can promote the regression of liver fibrosis by reducing the autophagy signaling pathway mediated by inflammation [5]. Ginsenoside Rg1 not only down-regulates GAS5 expression through GAS5/EZH2/SOCS3/NRF2, reduces microglial activation, and improves mitochondrial dysfunction in depression but also alleviates acute ulcerative colitis by regulating the intestinal flora and microbial tryptophan metabolism [6,7]. In addition, ginsenoside Rg1 significantly alleviated neuronal injury by reducing the levels of IL-6, IL-1β, and ROS and inhibiting the AIM2 inflammasome in rat brains, while ginsenoside Rg5 was able to reduce the levels of inflammatory cytokines TNF-α and IL-1β and improve cognitive deficits in a dose-dependent manner [8,9].

Alzheimer’s disease (AD) is an age-related degenerative disease of the central nervous system, characterized by amyloid plaques and Tau tangles. According to Alzheimer’s Disease International (ADI), there are at least 50 million dementia patients worldwide. This number is expected to reach 152 million by 2050 [10]. Studies have shown that AD patients not only produce Aβ protein deposition and Tau protein phosphorylation in brain tissue but also suffer from oxidative stress, apoptosis, neuroinflammation, neurovascular injury, neurotransmitter disorders, and mitochondrial dysfunction in the body [11]. However, studies have shown that ginseng can be used to treat Alzheimer’s disease by regulating neurotransmitter disorders, energy metabolism disorders, and neuroprotection. Ginsenosides Rg1 and Rh2 can promote neurite growth, reduce hippocampal damage, and improve cognitive levels through the PI3K/AKT/GSK-3β pathway mediated by antioxidant, anti-inflammatory, and anti-apoptotic effects [12,13,14]. Studies have also shown that physical exercise regulates brain function. Moreover, exercise may play a protective role in brain aging by increasing cerebral blood flow, thereby reducing inflammation, Aβ formation, and the phosphorylated Tau protein in the cerebral cortex and subcutaneous area. At the same time, the APOE gene is involved in the lipid metabolism and transport of proteins in the central nervous system, promoting lipid droplet formation and cholesterol accumulation in astrocytes, providing nutrition to neurons, and supporting the structure of the brain [15,16]. Ginsenoside Rg1 can effectively improve scopolamine-induced memory disorders and cognitive deficits by inhibiting decreases in 5-hydroxytrypsin and the activity of acetylcholinesterase [17]. Moreover, ginsenoside RK3 promotes neurogenesis and synaptogenesis through the CREB/BDNF signaling pathway, thereby promoting learning and cognition in AD mice [18].

Metabolomics has been widely used to study cerebrospinal fluid, blood (serum and plasma), urine, and brain tissue in AD. A combination of metabolomics and network pharmacology, along with metabolome and genome-wide association analysis, could help realize high-throughput screening of endogenous metabolite changes and identify relevant metabolic pathways related to the treatment of AD. Metabolomics analysis showed that ABCA1 and CPT1A are involved in regulating acylcarnitines and amino acids in AD. It can also reverse disorders of the intestinal flora and increase the content of SCFAs produced by the body by regulating endogenous metabolites [19,20,21,22]. With its high-throughput detection capabilities and complete data analysis tools, metabolomics is widely used to analyze metabolite levels in cells, tissues, and body fluids, thereby revealing the various metabolic networks and their relationship to biological functions.

In this study, PC12 cell model activity screening combined with metabolomics methods were used to explore GSF’s mechanism of action on AD. Firstly, the anti-AD activity of GSF in vitro was investigated using the Aβ25–35-induced PC12 cell AD model. Then, an AD mice model was used to explore the related metabolic pathways and potential therapeutic biomarkers of GSF against AD based on the metabolomics analysis.

## 2. Materials and Methods

### 2.1. Preparation of Ginsenoside

The origin of forest-grown ginseng (GSF) is Jilin Province, China. This variety of ginseng was first identified by Professor Shumin Wang of the Changchun University of Chinese Medicine. In this study, backflow extraction was carried out with 85% ethanol–aqueous solution at a 1:20 (*m*:*v*) liquid–solid ratio. The extraction solution was extracted four times, combined, and concentrated. The extraction solution was redissolved in water, eluted with 75% ethanol, and purified with D101 macroporous resin. Purified samples were then collected and freeze-dried.

### 2.2. Chemicals and Materials

Rat adrenal pheochromocytoma cells (PC12 cells) were purchased from Shanghai Zhong Qiao Xin Zhou Biotechnology Co., Ltd. (Shanghai, China). Enzyme-linked immunosorbent assay kits of interleukin (IL)-8 and superoxide dismutase (SOD) were purchased from Hushi Kenke Biotechnology Co., Ltd. (Huangshi, China). The basic medium, Phosphate Buffer Saline (PBS), and pancreatic enzyme were purchased from Shanghai beyotime Biotechnology Co., Ltd. (Shanghai, China). Acetonitrile, methanol, and formic acid used in this study were purchased from Fisher Scientific (Fairlawn, NJ, USA). Deionized water for the mass spectrometry was prepared using the Milli-Q Water Purification System (Milford, MA, USA). All other reagents used in this study were of analytical purity and purchased from Beijing Chemical Reagent Research Institute Co., Ltd. (Beijing, China). D-(+)-Galactose and the standards were purchased from Shanghai Yuanye Bio-Technology Co., Ltd. (Shanghai, China). Donepezil Hydrochloride tablets were produced by Eisai China Inc. (Suzhou, China).

### 2.3. PC12 Cells for the Experiments

#### 2.3.1. Evaluating the Optimal Concentrations of GSF and Aβ25–35

PC12 cells were cultured in an RPMI-1640 medium supplemented with 10% fetal bovine serum, 100 U/mL of streptomycin, and 100 U/mL of penicillin in an incubator at 37 °C with 5% CO_2_.

PC12 cells were inoculated into 96-well cell culture plates at a density of 1 × 10^4^/mL and cultured overnight in a 37 °C, 5% CO_2_ incubator. The cells were then treated with different concentrations of Aβ25–35 (5, 10, 20, 40, 80, 100, and 200 μmol/L) and GSF (10, 50, 100, 200, 400, 800, and 1000 μg/mL) for 24 h, and 6 wells were subjected to repeated tests for each concentration. After 24 h, the culture medium was discarded and supplemented with 100 μL of the culture medium containing 10% CCK-8. The absorbance was measured at 450 nm after incubation at 37 °C and with 5% CO_2_ in an incubator for 2 h. The optimal concentration of Aβ25–35 was determined by the cell inhibition rate, and the optimal concentration of GSF was determined by the cell proliferation rate.

CCK-8 was used to screen the concentration of forest-grown ginseng and Aβ25–35 and, thus, explore the effect of forest-grown ginseng on the cell viability of the PC12 cell. The cell proliferation rate was calculated using the following formula:

Proliferation rate% = [(ODs − ODb)/(ODc − ODb)] × 100%(1)

where ODs refers to the absorbance of the experimental group; ODb is the absorbance of the blank control; and ODc refers to the absorbance of the control group.

#### 2.3.2. Determination of the Oxidative Factor and Inflammatory Factor

PC12 cells were inoculated in 6-well plates at a density of 2.5 × 10^5^ cells/well during the logarithmic growth period and cultured in a 5% CO_2_ incubator at 37 °C for 24 h. The experimental groups were the CON group, MOD group, and GSF group (200 μg/mL, 100 μg/mL, 50 μg/mL). The cells and cell supernatant were collected according to the requirements of the kit, and the samples were treated according to the instructions of the kit. The concentrations of IL-8 and SOD in the cells were detected using an ELISA kit.

### 2.4. Animals and Experimental Design

The C57BL/6 mice (17 ± 0.1 g) used in the experiments were purchased from Liaoning Changsheng Biotechnology Co., Ltd., (Shenyang, China) (SCXK (Liao) 2020-0001). The Institutional Animal Care and Use Committee of the Changchun University of Chinese Medicine authorized the animal experiments with permission number CPCCUCM IACUC2022517. Mice were group-housed in cages under standard breeding conditions (12:12 h light–dark cycle, a controlled room temperature of 23 °C ± 2 °C, sanitary conditions, and a standard diet). After allowing 1 week for acclimation, mice were randomly divided into 6 groups with 12 mice in each group. To investigate whether the therapeutic effect of GSF on AD is dose-dependent, different concentrations of GSF were established. The groups were defined as follows: a control group (CON group); model group (MOD group); donepezil group (reference drug, POS group, 1.0 mg/kg/day); low-dose GSF group (LGS group, 1.5 g/kg/day); medium-dose GSF group (MGS group, 2.25 g/kg/day); and a high-dose GSF group (HGS group, 3.0 g/kg/day). According to the Chinese Pharmacopoeia, the daily dosage for people is 3~9 g. The equivalent dose for mice was determined by converting the body surface area. All GSF doses and donepezil were administered for 8 weeks via gavage. All groups except for the CON group received intraperitoneal injections of D-Gal (1000 mg/kg/day) and AlCl_3_ (10 mg/kg/day) for 8 weeks. The mice were given only water 12 h before the last dose, and drugs 2 h before dissection. The mice were killed via dislocation of the neck after collecting blood by removing the eyeballs. The brain tissue was separated and collected on ice. Part of the brain tissue was kept at −80 °C for further analysis of the pathological section and ELISA determination. Other portions were fixed in 10% paraformaldehyde and sectionalization for immunohistochemical analysis.

The collected eyeball blood was allowed to stand for 60 min at room temperature, then it was centrifuged at 13,000 r/min at 4 °C. Afterward, the upper serum was stored at −80 °C, which naturally melted at room temperature when performing the metabolomics analyses. A total of 100 µL of serum was vortexed with 300 µL of ice-cold methanol for 2 min and centrifuged at 12,000 r/min for 15 min at 4 °C. Then, the supernatant was blown dry with nitrogen, redissolved with 200 µL of methanol, passed through a 0.22 µM filter membrane, and analyzed using a UHPLC-Q-Exactive MS.

In total, 500 mg of mouse brain tissue was collected and homogenized with a precooled organic solution (methanol–acetonitrile–water = 2:2:1) at a ratio of 1:3. The homogenate was centrifuged at 12,000 rpm for 10 min at 4 °C, and the supernatant was blown dry with nitrogen gas. Then, 100 µL of aqueous acetonitrile solution (acetonitrile–water = 1:1) was dissolved, passed through a 0.22 µM filter membrane, and analyzed using the UHPLC-Q-Exactive MS.

### 2.5. Serum UHPLC-MS Metabolomic Analysis

Raw metabolomics data were acquired with the UHPLC-Q-Exactive MS system (Thermo, Waltham, MA, USA). A Thermo Syncronis C18 column (100 mm × 3.0 mm × 1.7 μm) was used for metabolite separation. Throughout the analysis, the column and sample chamber temperatures were maintained at 25 °C and 4 °C, respectively. The optimal mobile phases were 0.1% (*v*/*v*) formic acid in water (A) and acetonitrile (B). The optimized gradient was as follows: 0~1 min: 0~3%B; 1~16 min: 3~70%B; 16~18 min: 70%B; 18~25 min: 70~90%B; 25~26 min: 90%B; 26~30 min: 90~100%B; 30~30.5 min: 100~90%B; 30.5~31 min: 90~70%B; 31~31.5 min: 70~3%B; 31.5~32 min: 3~0%B. The flow rate was set to 0.30 mL/min and the injection volume was 2.0 μL.

The metabolites were detected in both negative- and positive-ion modes using a mass spectrometer with an electron spray ionization (ESI) source. The MS parameters were as follows: capillary voltage, 3.0~2.5 kV; desolvation temperature, 350 °C; desolvation gas flow rate, 600 L/h; source temperature, 120 °C; sampling cone, 40 V; cone gas flow rate, 50 L/h; MSE mode; low collision energy, 7 eV; high collision energy, 10~35 eV; mass range, *m*/*z* 50~1000.

### 2.6. Brain Tissue UHPLC-MS Metabolomic Analysis

Raw metabolomics data were acquired with the UHPLC-Q-Exactive MS system (Thermo, Waltham, MA, USA). The Thermo Syncronis C18 column (100 mm × 3.0 mm × 1.7 μm) was used for metabolite separation. Throughout the analysis, the column and sample chamber temperatures were maintained at 25 °C and 4 °C, respectively. The optimal mobile phases were 0.1% (*v*/*v*) formic acid in water (A) and acetonitrile (B). The optimized gradient was as follows: 0~6 min: 80%B; 6~7 min: 80~95%B; 7~8 min: 95%B; 8~8.5 min: 95~75%B; 8.5~9 min: 75~20%B; 9~9.5 min: 20%B; 9.5~10 min: 20~80%B; and 10~12 min: 80%B. The flow rate was set to 0.30 mL/min and the injection volume was 2.0 μL.

The metabolites were detected in both negative- and positive-ion modes using a mass spectrometer with an electron spray ionization (ESI) source. The MS parameters were as follows: capillary voltage, 3.0~2.5 kV; desolvation temperature, 350 °C; desolvation gas flow rate, 600 L/h; source temperature, 120 °C; sampling cone, 40 V; cone gas flow rate, 50 L/h; MSE mode; low collision energy, 7 eV; high collision energy, 10~35 eV; mass range, *m*/*z* 100~1500.

### 2.7. Statistical Analysis

Raw data files from the UHPLC-Q-Exactive Orbitrap MS were processed using the Thermo Sieve v2.0 software (Waltham, MA, USA). The retention time, mass charge ratio, and peak area normalization were calculated. After processing with the Sieve software, all compound information was imported into SIMCA 14.1 for the multivariate statistical analysis, including principal component analysis (PCA) and orthogonal partial least squares discrimination analysis (OPLS-DA), which were used to identify differential ions. The HMDB database (https://www.hmdb.ca/, accessed on 26 December 2024) was used to search candidate metabolites and identify potential marker information. KEGG (https://www.kegg.jp/, accessed on 26 December 2024) and MetaboAnalyst (https://www.metaboanalyst.ca/, accessed on 26 December 2024) were used to analyze relevant metabolites and metabolic pathways.

## 3. Results

### 3.1. Protective Effect of GSF on PC12 Cell Injury Induced by Aβ25–35

#### 3.1.1. Effect of GSF on PC12 Cell Proliferation

**Figure 1:**
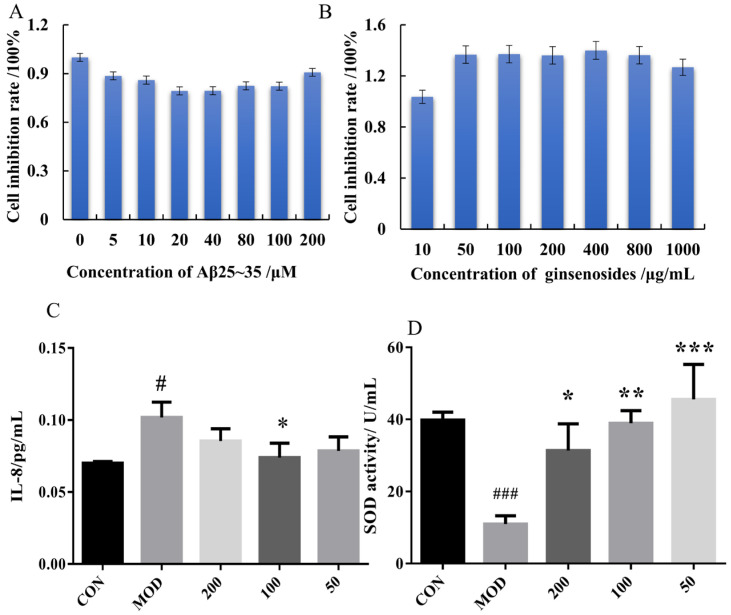
Effects of GSF on the cell viability of PC12 cells (*n* = 3): (**A**,**B**) effects of Aβ25–35 and GSF on CCK-8; (**C**,**D**) effects of GSF on cell IL-8 and SOD secretion vs. CON group, # *p* < 0.05 and ### *p* < 0.001, and vs. MOD group, * *p* < 0.05, ** *p* < 0.01, *** *p* < 0.001.

To explore the effect of GSF on the proliferation of PC12 cells, the toxic effects of GSF on PC12 cells were evaluated via cytotoxicity assays. At the same time, the optimal dose of Aβ25–35 protein to induce the AD model in PC12 cells was investigated in a liquid, as shown in Figure 1A,B. The optimal concentration of Aβ25–35 was 20 μM. GSF has no obvious toxicity to PC12 cells in the range of 50–800 ug/mL, and exerts a promoting effect on PC12 cells to a certain extent. The optimal concentration of GSF is 50–200 μg/mL. Therefore, the concentration range of GSF was set between 50 and 200 μg/mL in the subsequent experiments.

#### 3.1.2. Pharmacodynamic Evaluation of PC12 Cells

SOD plays an important role in the balance of cellular oxidation and resistance to hydrogenation. Figure 1C shows that the mass concentration of SOD in PC12 cells in the model group was significantly decreased (*p* < 0.05) compared with that in the CON group (no medical induction or treatment). The concentration of SOD in the PC12 cells of the model group was lower than that of the control group, indicating that the Aβ25–35 protein could reduce the mass concentration of SOD in cells, thereby decreasing SOD activity. This phenomenon is due to the significant production of lipid peroxides after treatment with the Aβ25–35 protein, which inhibits SOD activity. However, the intracellular SOD concentration of cells pretreated with different concentrations of GSF was significantly higher than that of the model. After PC12 cells were injured by Aβ25–35, the levels of IL-8 secreted by PC12 cells increased. However, the levels of IL-8 secreted by PC12 cells decreased after GSF intervention (*p* < 0.01). GSF reduced the levels of inflammatory factors in PC12 cells and alleviated the inflammatory response.

### 3.2. Immunohistochemical Results

**Figure 2:**
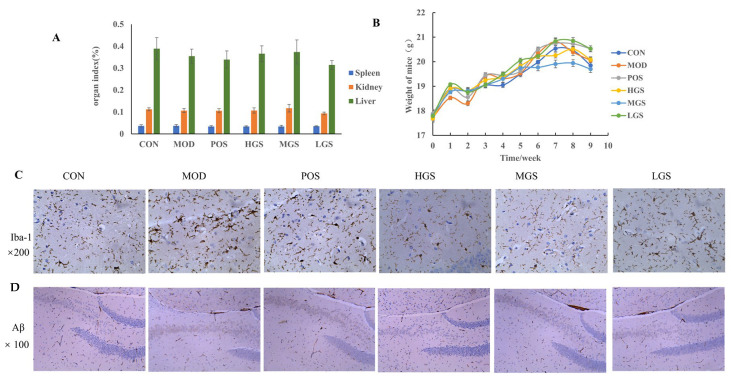
Body weight and organ indexes of the mice and immunohistochemical results of brain tissue: (**A**) weight changes; (**B**) organ index; (**C**) immunohistochemical analysis of IBa1 (×200); (**D**) Aβ deposition in the hippocampus of the mice (×100). The pictures in (**C**) from left to right are CON, MOD, POS, HGS, MGS, and LGS groups, respectively. The (×100) and (×200) in the figure means the field of view 100 and 200 times larger under the inverted microscope.

Body weight and organs (including the spleen, kidney, and liver) were evaluated. The results in Figure 2A,B indicate no statistically significant differences in the body weight or organ indexes among the groups. This result demonstrates that establishing an AD model and administering GSF had significant effects on the body weights of mice, confirming that the dose of GSF was within a reasonable range. The deposition of Aβ can activate astrocytes and microglia and destroy the blood–brain barrier, which is the main cause of neuronal degeneration and death in AD patients. Ionized calcium-binding adaptor molecule 1 (Iba1) is a calcium-binding protein specific for microglia and macrophages, which participates in the formation of cell membrane folds and the phagocytosis of activated microglia. Immunohistochemical staining of Iba1 revealed large numbers of deeply stained brown Iba1-positive cells in the mice brain sections of the MOD group. Here, the cell morphology was clear: the cell bodies increased in size, the process became thicker, and the number of branch networks significantly increased. These results were significantly different from that of the CON group and the treated group. Compared with that of the MOD group, the response of the GSF group was weaker, the Iba1 staining was lighter, the cell morphology became blurred, and the number of positive cells in the branching network decreased (Figure 2C). The Aβ immunohistochemical results are shown in Figure 2D. Here, the color yellow indicates beta-amyloid deposits. There was a more positive expression in the MOD group compared with the results in the CON and treated groups. The accumulation of Aβ protein was obvious. However, after different doses of GSF intervention, the number of yellow particles decreased to different degrees. This result indicates that GSF can significantly improve Aβ deposition.

### 3.3. UHPLC-Q-Exactive MS-Based Serum Metabolomics Analysis

**Figure 3:**
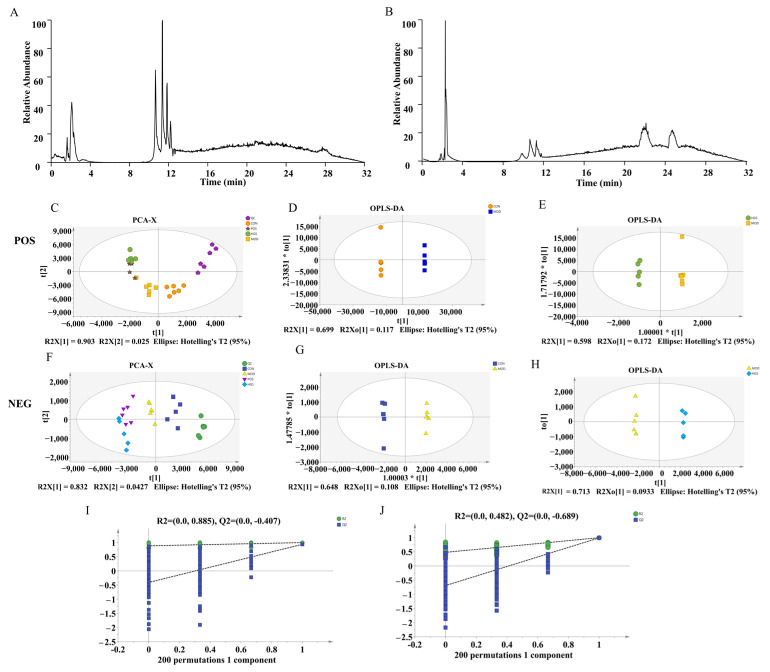
Endogenous metabolites changed in AD mice serum: (**A**,**B**) serum metabolic profiling of the MOD group in the negative- and positive-ion modes of UHPLC-Q-Exactive MS; (**C**–**H**) PCA score plots and OPLS-DA score plots of the MOD vs. CON and HGS vs. MOD groups in the positive and negative models; (**I**,**J**) permutation plot for 2 groups using the 200-response reciprocity test in the positive and negative modes for the MOD vs. HGS groups.

Based on the pathological results, the serum metabolic profiles of mice were analyzed using UHPLC-Q-Exactive MS to explore the neuroprotective mechanism of ginsenoside. The positive and negative basal peak intensity (BPI) chromatograms of serum samples from the MOD group are shown in Figure 3A,B. To ensure the stability and reliability of the system and data, quality control (QC) samples were tested during the analysis. The QC samples clustered together, indicating the stability and reproducibility of the instrumental analysis. Then, we performed unsupervised principal component analysis (PCA) and orthogonal partial least square discriminate analysis (OPLS-DA) to evaluate the clustering trends. The positive- and negative-ion patterns of the PCA, OPLS-DA plots, and S-plots are shown in Figure 3C–J. Generally, when the value of Q2 > 0.5, the model is more stable and reliable. The parameters R2X = 0.928 and Q2 = 95% for the AD and CON groups in the positive-ion mode, respectively, and R2X = 0.875 and Q2 = 95% for the AD and CON groups in the negative-ion mode, indicated significant differences in endogenous metabolite levels in the AD group compared with the CON group. These results also indicated that the model was successfully established. According to the OPLS-DA score plot, HGS and MOD exhibited separation in the positive and negative models demonstrating metabolite differences between the two groups. After further construction and differentiation via supervised orthogonal partial least squares discriminant analysis (OPLS-DA), different separation clusters could be observed in the OPLS-DA score map (OPLS-DA score plots). Then, the model was iterated 200 times with a Permutation test. The intercepts of the regression line at point Q2 were −0.728 and −0.689, respectively. All the R2 values and Q2 values on the left are lower than the original values on the right, which demonstrates that the model does not produce overfitting and is both stable and reliable.

**Figure 4:**
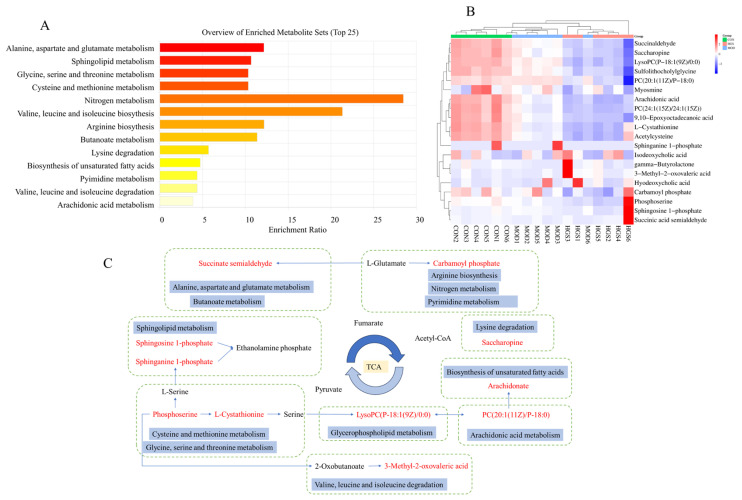
Heatmap and enrichment pathway analysis of serum metabolomic differential metabolites: (**A**) metabolic pathway enrichment analysis; The column length represents the number of potential biomarkers enriched into the pathway, and the red depth represents the significance of the *p*-value. (**B**) heatmap showing the intensities of the potential biomarkers in each group; and (**C**) the correlation networks between the main differential metabolites and the corresponding metabolic pathways. The arrows between potential biomarkers represent the direction of the effect, which includes promotion or inhibition.

The OPLS-DA results and VIP values of the corresponding metabolites were combined to screen and extract the metabolites associated with the HGS regulation of AD (VIP > 1 and *p* < 0.05). Then, an analysis was carried out to visualize the up-regulated (FC > 1.0) and down-regulated expressions (FC < 1.0). A univariate statistical RT analysis, accurate MS, and differential compounds were introduced into the metabolomics database HMDB (https://hmdb.ca/spectra/ms/search, accessed on 11 August 2024.). The ion intensities of 14 metabolites between the GSH group and MOD group were used to construct a heatmap. The expression of metabolites was significantly different between these groups, as shown in Figure 4A. To investigate possible metabolic pathways in the HGS treatment of AD mice, 14 metabolic pathway biomarkers were enriched and topologically analyzed using the MetaboAnalyst 5.0 metabolic option. The MS/MS information for these biomarker metabolites is shown in Table 1. The KEGG pathway enrichment analysis of the differential metabolites showed that 20 differential metabolites were enriched in 13 metabolic pathways (Figure 4B). These metabolic pathways mainly included amino acid metabolism, sphingolipid metabolism, unsaturated fatty acid metabolism, primary bile acid metabolism, pyrimidine metabolism, and nitrogen metabolism.

### 3.4. UHPLC-Q-Exactive MS-Based Brain Tissue Metabolomics Analysis

**Figure 5:**
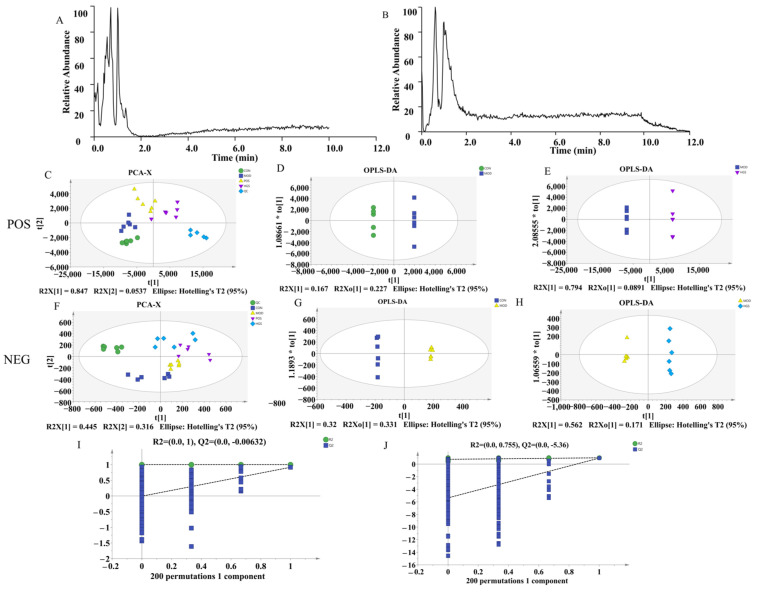
Endogenous metabolites changed in AD mice brains: (**A**,**B**) brain metabolic profiling of the MOD group in the negative- and positive-ion modes of the UHPLC-Q-Exactive MS; (**C**–**H**) PCA score plots and OPLS-DA score plots of the MOD vs. CON and HGS vs. MOD groups in the positive and negative models; (**I**,**J**) permutation plot for 2 groups using the 200-response reciprocity test in the positive and negative modes for the MOD vs. HGS groups.

The brain tissue metabolic profiles of mice were analyzed via UHPLC-Q-Exactive MS to explore the neuroprotective mechanism of ginsenoside. The positive and negative BPI chromatograms of brain samples from the MOD group are shown in Figure 5A,B. PCA and OPLS-DA were used to evaluate cluster trends. The positive- and negative-ion patterns of the PCA, OPLS-DA plots, and S-plots are shown in Figure 5C–J. The QC samples clustered together, indicating good stability and reproducibility of the instrumental analysis. The parameters R2X = 0.928 and Q2 = 95%, respectively, for the AD and CON groups in the positive-ion mode and R2X = 0.875 and Q2 = 95% for the MOD and CON groups in the negative-ion mode indicated significant differences in endogenous metabolite levels in the MOD group compared with those in the CON group. Thus, the model was successfully established. According to the OPLS-DA score plot, HGS and MOD exhibited separation in the positive and negative models, demonstrating metabolite differences between the two groups. Then, the model was iterated 200 times with a Permutation test. The intercepts of the regression line at point Q2 were −0.728 and −0.689, respectively. All the R2 values and Q2 values on the left are lower than the original values on the right, which indicates that the model did not produce overfitting, has good stability, and is reliable.

**Figure 6:**
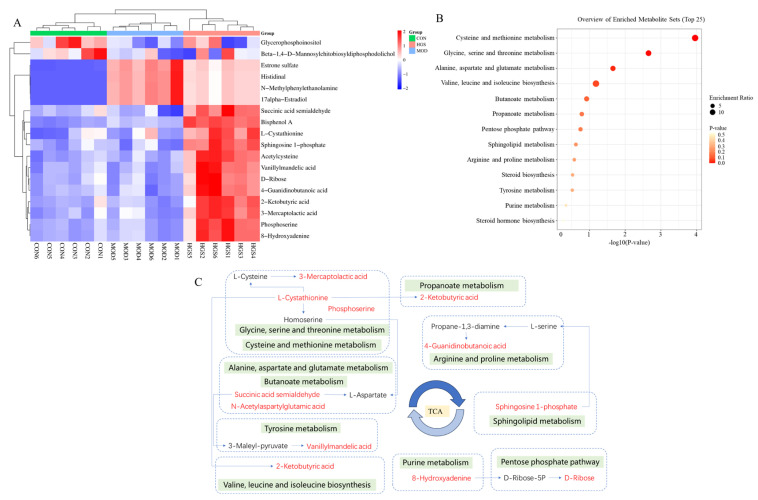
Heatmap and enrichment pathway analysis of mice brain tissue metabolomic differential metabolites: (**A**) heatmap showing the intensities of the potential biomarkers in each group; (**B**) metabolic pathway enrichment analysis; and (**C**) the correlation networks between the main differential metabolites with the corresponding metabolic pathways. The arrows between potential biomarkers represent the direction of the effect, which includes promotion or inhibition.

Multiple groups were compared to search for specific metabolic biomarkers. Both *p* < 0.05 and VIP > 1.0 were considered statistically significant. The OPLS-DA results and VIP values of the corresponding metabolites were combined to screen and extract the metabolites associated with the HGS regulation of AD (VIP > 1.0 and *p* < 0.05). Then, an analysis was carried out to visualize the up-regulated (FC > 1.0) and down-regulated expressions (FC < 1.0). A univariate statistical RT analysis, accurate MS, and differential compounds were introduced into the HMDB metabolomics database (https://hmdb.ca/spectra/ms/search, accessed on 1 August 2024.). The potential biomarkers were used to construct a heatmap, and the expression of metabolites was significantly different between the groups, as shown in Figure 6A. To investigate the possible metabolic pathways in the HGS treatment of AD mice, the biomarker metabolic pathways were enriched and topologically analyzed using the MetaboAnalyst 5.0 metabolic option. The above data and MS/MS information for these biomarker metabolites are shown in Table 2. There were six potential biomarkers for metabolites in the positive- and negative-ion modes in the CON and MOD groups, and twelve potential biomarkers for metabolites in the positive- and negative-ion modes in the MOD and HGS groups.

The KEGG pathway enrichment analysis of differential metabolites showed that the differential metabolites were enriched in 13 metabolic pathways (Figure 6B): cysteine and methionine metabolism; glycine, serine, and threonine metabolism; alanine, aspartate, and glutamate metabolism; valine, leucine, and isoleucine biosynthesis; butanoate metabolism; propanoate metabolism; the pentose phosphate pathway; sphingolipid metabolism; arginine and proline metabolism; steroid biosynthesis; tyrosine metabolism; Purine metabolism; and steroid hormone biosynthesis. A schematic representation of the metabolites and related pathways based on these results is shown in Figure 6C. In total, 11 regulated metabolic pathways and 12 key differential metabolites are involved.

## 4. Discussion

Because the deposition of Aβ protein plays an important role in the pathogenesis of AD, this phenomenon represents an important cause of nerve cell death and the destruction of the blood–brain barrier [23,24]. PC12 cells have neuronal properties and can be induced by the Aβ25–35 protein to produce pathological features related to AD. Therefore, using in vitro experiments, PC12 cells were induced by the Aβ25–35 protein to construct an AD model on PC12 cells. The results showed that GSF can increase SOD content, enhance the removal of superoxide free radicals, prevent the production of hydroxyl free radicals, and avoid oxidative stress. Moreover, GSF plays a neuroprotective role by reducing IL-8 and alleviating the inflammatory response caused by IL-8 abnormalities.

GSF’s mechanism of action in the treatment of Alzheimer’s disease was also studied through in vivo experiments. IBa1 is a calcium-binding protein specifically expressed in the microglia of the central nervous system. This protein is involved in the cell membrane folding and phagocytosis of activated microglia and plays an important role in the pathological process of AD. However, overexpression of Iba1 can aggravate inflammatory responses, cause microglia to phagocytose neurons or release chemokines to produce neurotoxicity, destroy the integrity of the blood–brain barrier, and aggravate the process of AD [25,26,27]. GSF can enhance the phagocytosis of the Aβ protein by regulating the activation state of the microglia and reduce the damage to neurons caused via inflammatory responses by inhibiting the over-activation of the microglia. This activity is further supported by GSF’s ability to improve the pathological injury of AD mice brain tissue by reducing the deposition of the Aβ protein induced via inflammation.

Studies have shown that the development of AD interferes with a variety of endogenous metabolite biomarkers [28]. Serum and brain tissue metabolomics were used to explore the regulatory mechanisms of endogenous metabolites during the GSF retreatment of AD. Serum metabolomics analysis revealed 20 potential differential metabolites that were mainly related to nitrogen metabolism, amino acid metabolism, and sphingolipid metabolism. Metabolomics analysis of the brain tissue identified 17 potential biomarkers, which affected 13 metabolic pathways, including the metabolism and synthesis of neurotransmitters; butanoate metabolism; propanoate metabolism; the pentose phosphate pathway; and sphingolipid metabolism. Succinic acid semialdehyde is a substrate of gamma-aminobutyric acid (GABA), a major inhibitory neurotransmitter in the brain. This acid can improve disorders of the neurotransmitter metabolic pathway, regulate neural activity, activate brain glucose metabolism, promote acetylcholine synthesis, and improve AD [29]. By regulating changes in the content of arachidonic acid—the most widely distributed endogenous active substance in the body—metabolic disorders of unsaturated fatty acids can be ameliorated, the natural physiological functions of the body can be maintained, and the inflammatory response of the body can be alleviated [30]. Sphingosine 1-phosphate (S1P) is a key pathway in sphingolipid metabolism involved in the regulation of cell proliferation, survival, and apoptosis. Restoring the content of S1P in AD mice can regulate metabolic disorders, improve the functions of body cells, alleviate neuroinflammation caused by abnormal content in brain tissue, change the proteolytic activity of β-secrease BACE1, control the production of Aβ protein, and alleviate AD [31]. L-cysteine is a combination of homocysteine and serine. This amino acid is mainly used as an intermediate in the metabolism of sulfur-containing amino acids and participates in complex metabolic processes. GSF can affect the cysteine and methionine metabolic pathways by causing content changes in L-Cystathionine, 2-Ketobutyric acid, Phosphoserine, and 3-Mercaptolactic acid [32,33]. These metabolic pathways affect the TCA cycle either directly or indirectly. The TCA cycle is an important hub for the metabolism of carbohydrates, fats, and proteins and provides energy for the body. Therefore, GSF regulates endogenous substances to inhibit the expression of Iba1, thereby inhibiting the release of inflammatory factors and alleviating inflammatory responses.

## 5. Conclusions

In this study, untargeted serum and brain tissue metabolomics combined with PC12 cell activity were successfully used to investigate treatments for AD. The results showed that GSF restored the levels of inflammatory cytokine IL-8 and oxidation cytokine SOD in PC12 cells in vitro, suggesting that GSF can reduce neuroinflammation and oxidative stress. The experimental results in mice showed that GSF can improve neurotransmitter metabolism and energy metabolism disorders, restore neurotransmitter levels, reduce Aβ protein deposition, reduce inflammatory factor overexpression, and reduce nerve cell damage, thus playing an anti-AD role, providing a theoretical basis for the development of ginseng products in the treatment of AD.

## Figures and Tables

**Table 1:** Potential biomarkers for UHPLC-MS/MS identification in serum.

NO.	Mass	Compound_id	Name	Formula	Adduct	Adduct Type	Rt/ min	MS/MS	KEGG	Change Trend		VIP		FC		*p*-Value	
										MOD/CON	HGS/MOD	MOD/CON	MOD/HGS	CON/MOD	MOD/HGS	CON/ MOD	MOD/ HGS
1	155.0516	HMDB0000272	Phosphoserine	C_3_H_8_NO_6_P	M+3ACN+2H	POS	12.70	98.9874, 113.9670, 131.9778	C01005	↓	↑	1.06	1.29	0.66	1.20	5.75 × 10^−3^	4.78 × 10^−3^
2	173.0808	HMDB0258537	Succinaldehyde	C_4_H_6_O_2_	2M+H	POS	3.44	149.9444, 112.0315, 84.0179	C16835	↓	↓	1.28	1.75	0.64	0.79	7.35 × 10^−5^	1.35 × 10^−3^
3	173.0808	HMDB0000549	gamma-Butyrolactone	C_4_H_6_O_2_	2M+H	POS	10.90	112.0315, 109.1046, 84.0179	C01770	↑	↓	1.32	1.69	1.29	0.83	1.08 × 10^−5^	6.89 × 10^−3^
4	183.0165	HMDB0001096	Carbamoyl phosphate	CH_4_NO_5_P	M+ACN+H	POS	13.04	155.0510, 127.0191, 98.9874	C00169	↓	↑	1.05	1.18	0.65	1.19	6.51 × 10^−3^	1.51 × 10^−2^
5	205.0647	HMDB0000099	L-Cystathionine	C_7_H_14_N_2_O_4_S	M+H−H_2_O	POS	2.42	162.8056, 123.1631, 89.5861	C02991	↑	↓	1.30	1.14	1.26	0.62	2.19 × 10^−5^	1.35 × 10^−3^
6	205.0641	HMDB0001890	Acetylcysteine	C_5_H_9_NO_3_S	M+ACN+H	POS	12.67	184.1715, 166.5062, 89.5861	C06809	↓	↑	1.05	1.16	0.70	1.29	4.50 × 10^−4^	4.00 × 10^−3^
7	246.0972	HMDB0001259	Succinic acid semialdehyde	C_4_H_6_O_3_	2M+ACN+H	POS	2.64	206.0689, 205.0656, 145.3397	C00232	↑	↑	0.18	0.96	1.18	0.80	1.48 × 10^−2^	3.96 × 10^−2^
8	261.1333	HMDB0000491	3-Methyl-2-oxovaleric acid	C_6_H_10_O_3_	2M+H	POS	1.70	218.9760, 162.7912, 97.6987	C00671	↑	↑	1.29	1.80	1.23	0.61	3.08 × 10^−5^	5.41 × 10^−4^
9	266.1586	HMDB0002639	Sulfolithocholylglycine	C_26_H_43_NO_7_S	M+H+NH4	POS	16.62	265.1372, 246.1556, 135.0265	C11301	↓	↓	1.28	1.31	0.86	0.86	4.83 × 10^−4^	2.13 × 10^−3^
10	277.1394	HMDB0000279	Saccharopine	C_11_H_20_N_2_O_6_	M+H	POS	6.83	214.0946, 183.0825, 112.0312	C00449	↑	↑	1.25	1.51	1.09	1.49	1.19 × 10^−6^	2.11 × 10^−3^
11	346.2741	HMDB0001043	Arachidonic acid	C_20_H_32_O_2_	M+ACN+H	POS	18.12	284.3022, 162.8419, 88.0788	C00219	↑	↓	1.26	1.80	1.59	0.52	2.89 × 10^−6^	5.82 × 10^−5^
12	362.2461	HMDB0000277	Sphingosine 1-phosphate	C_18_H_38_NO_5_P	M+H−H_2_O	POS	2.45	249.7067, 162.7943, 137.4978	C06124	↓	↑	1.01	1.21	0.69	1.20	5.24 × 10^−4^	1.47 × 10^−2^
13	421.3433	HMDB0008325	PC(20:1(11Z)/P-18:0)	C_46_H_90_NO_7_P	M+ACN+2H	POS	1.86	414.4126, 213.8097, 162.7904	C00157	↑	↑	1.07	1.29	1.39	1.38	6.23 × 10^−4^	4.93 × 10^−4^
14	453.3581	HMDB0002536	Isodeoxycholic acid	C_24_H_40_O_4_	M+IsoProp+H	POS	2.93	435.3441, 322.2569, 226.1973	C17661	↓	↑	1.13	1.56	0.88	1.15	1.23 × 10^−3^	3.50 × 10^−4^
15	453.3583	HMDB0000733	Hyodeoxycholic acid	C_24_H_40_O_4_	M+IsoProp+H	POS	10.58	413.2768, 209.1705, 114.0947	C15517	↓	↑	1.21	1.40	0.90	1.09	1.63 × 10^−3^	7.57 × 10^−3^
16	498.4112	HMDB0008816	PC(24:1(15Z)/24:1(15Z))	C_56_H_108_NO_8_P	M+ACN+2H	POS	10.65	435.3445, 322.2572, 209.1704	C00157	↓	↓	1.25	1.78	0.67	0.76	2.34 × 10^−5^	8.89 × 10^−4^
17	588.4136	HMDB0010408	LysoPC(P-18:1(9Z)/0:0)	C_26_H_52_NO_6_P	M+2ACN+H	POS	11.31	570.4187, 414.3601, 219.1586	C04230	↓	↑	1.28	1.78	0.78	1.35	1.32 × 10^−5^	2.02 × 10^−3^
18	611.4882	HMDB0061650	9,10-Epoxyoctadecanoic acid	C_18_H_34_O_4_	2M+H−H2O	POS	11.17	588.4225, 283.7238, 183.0824	C19620	↓	↓	1.22	1.69	0.70	0.77	1.38 × 10^−5^	1.38 × 10^−3^
19	362.2496	HMDB0001383	Sphinganine 1-phosphate	C_18_H_40_NO5P	M−H_2_O−H	NEG	2.17	355.2077, 319.2307, 285.1143	C01120	↓	↓	1.67	1.61	0.76	0.73	3.11 × 10^−5^	1.16 × 10^−4^
20	437.2456	HMDB0030386	Myosmine	C_9_H_10_N_2_	3M−H	NEG	24.65	379.2371, 333.2316, 89.0235	C10160	↓	↓	1.65	1.70	0.87	0.87	1.46 × 10^−4^	2.22 × 10^−3^

Note: The levels of potential biomarkers were labeled with (↓) down-regulated and (↑) up-regulated.

**Table 2:** Potential biomarkers identified via UHPLC-MS/MS in mice brain tissue.

Group	NO.	Rt/min	Mass	MS/MS	Name	Formula	Adduct	Adduct Type	Delta /ppm	KEGG	VIP	*p*-Value	log_2_FC	Change Trend
CON/MOD	1	1.92	341.1657	205.0654, 182.0830, 155.0508	17alpha-Estradiol	C_18_H_24_O_2_	M+H+HCOONa	POS	19	C02537	1.34	2.27 × 10^−10^	4.00	↑
	2	1.42	137.0495	132.0804, 123.0587104.1101	Bisphenol A	C_15_H_16_O_2_	M+2Na	POS	20	C13624	1.14	5.16 × 10^−10^	0.12	↑
	3	1.56	371.1663	205.0653, 183.0829, 132.0803	Beta-1,4-D-Mannosylchitobiosyldiphosphodolichol	C_47_H_82_N_2_O_22_P_2_	M+2H+Na	POS	10	C05860	1.89	2.62 × 10^−1^	−0.05	↓
	4	1.9	342.169	340.2681, 183.0829, 155.0508	Histidinal	C_6_H_9_N_3_O	2M+ACN+Na	POS	12	C01929	1.57	3.11 × 10^−10^	6.03	↑
	5	1.61	357.14	340.2680, 205.0653, 132.0803	Estrone sulfate	C_18_H_22_O_5_S	M+Li	POS	15	C02538	1.31	3.81 × 10^−10^	6.22	↑
	6	1.48	401.0543	303.0864, 292.9264, 174.0416	Glycerophosphoinositol	C_9_H_19_O_11_P	M−H+HCOONa	NEG	19	C01225	1.10	2.45 × 10^−3^	0.03	↑
MOD/HGS	1	1.58	183.0828	171.1536, 140.0144, 127.0189	D-Ribose	C_5_H_10_O_5_	M+CH_3_OH+H	POS	19	C00121	2.53	2.98 × 10^−4^	0.14	↑
	2	1.25	184.0861	146.0462, 132.0304, 124.0074	8-Hydroxyadenine	C_5_H_5_N_5_O	M+CH_3_OH+H	POS	17	C22499	1.31	2.59 × 10^−5^	0.14	↑
	3	1.55	205.0654	191.0209, 147.0302, 124.9914	Vanillylmandelic acid	C_9_H_10_O_5_	M+Li	POS	16	C05584	1.99	5.61 × 10^−5^	0.17	↑
	4	1.64	205.0654	174.0416, 133.0144, 110.9771	L-Cystathionine	C_7_H_14_N_2_O_4_S	M+H−H_2_O	POS	3	C02291	1.01	3.53 × 10^−4^	0.05	↑
	5	1.79	205.0654	194.9382, 138.9708, --	3-Mercaptolactic acid	C_3_H_6_O_3_S	M+2ACN+H	POS	6	C05823	3.09	2.65 × 10^−5^	0.17	↑
	6	3.49	228.1418	174.0032, 162.8690, 119.9471	4-Guanidinobutanoic acid	C_5_H_11_N_3_O_2_	M+2ACN+H	POS	16	C01035	1.09	7.23 × 10^−5^	0.11	↑
	7	8.74	246.0929	213.8909, 203.0141, 129.9759	Succinic acid semialdehyde	C_4_H_6_O_3_	2M+ACN+H	POS	18	C00232	1.24	9.60 × 10^−5^	0.08	↑
	8	4.9	246.0929	146.9665, 136.9090, 119.9470	2-Ketobutyric acid	C_4_H_6_O_3_	2M+ACN+H	POS	18	C00109	1.17	2.16 × 10^−4^	0.14	↑
	9	1.48	341.1657	320.0652, 304.0897, 285.0756	N-Methylphenylethanolamine	C_9_H_13_NO	2M+K	POS	9	C03711	1.17	2.27 × 10^−10^	0.19	↑
	10	1.98	362.2503	349.0920, 320.0652, 292.9264	Sphingosine 1-phosphate	C_18_H_38_NO_5_P	M+H−H_2_O	POS	12	C06124	1.14	2.46 × 10^−6^	0.12	↑
	11	1.23	155.0507	147.0499, 145.0622, 132.0304	Phosphoserine	C_3_H_8_NO_6_P	M+3ACN+2H	POS	6	C01005	1.80	2.63 × 10^−5^	0.15	↑
	12	1.48	401.0543	303.0864, 292.9264, 174.0416	Glycerophosphoinositol	C_9_H_19_O_11_P	M-H+HCOONa	NEG	19	C01225	1.10	2.45 × 10^−3^	0.09	↑

Note: The levels of potential biomarkers were labeled with (↓) down-regulated and (↑) up-regulated.

## Data Availability

Data are contained within the article.

## Abbreviations

The following abbreviations are used in this manuscript:

GSF	Forest-grown ginseng
SOD	Superoxide dismutase
AD	Alzheimer’s disease
PCA	Principal component analysis
UHPLC-Q-Exactive MS	Ultra-high-performance liquid chromatography–quadrupole-orbitrap high-resolution mass spectrometry
Aβ protein	Amyloid β-protein
